# Vibrational Stark Effect of the Electric-Field Reporter 4-Mercaptobenzonitrile as a Tool for Investigating Electrostatics at Electrode/SAM/Solution Interfaces

**DOI:** 10.3390/ijms13067466

**Published:** 2012-06-18

**Authors:** Gal Schkolnik, Johannes Salewski, Diego Millo, Ingo Zebger, Stefan Franzen, Peter Hildebrandt

**Affiliations:** 1 Technische Universität Berlin, Insitut für Chemie, Sekr. PC14, Straße des 17, Juni 135, Berlin, D-10623, Germany; E-Mails: gal.schkolnik@mail.tu-berlin.de (G.S.); johannes.salewski@tu-berlin.de (J.S.); d.millo@vu.nl (D.M.); ingo.zebger@tu-berlin.de (I.Z.); 2 Biomolecular Spectroscopy, LaserLaB Amsterdam, Vrije Universiteit Amsterdam, De Boelelaan 1083, Amsterdam, NL-1081 HV, The Netherlands; 3 Department of Chemistry, North Carolina State University, Box 8204, Raleigh, NC 27695, USA; E-Mail: stefan_franzen@ncsu.edu

**Keywords:** interfaces, self-assembled monolayer, electrode, vibrational Stark effect, surface enhanced Raman, surface enhanced infrared, potential of zero-charge

## Abstract

4-mercaptobenzonitrile (MBN) in self-assembled monolayers (SAMs) on Au and Ag electrodes was studied by surface enhanced infrared absorption and Raman spectroscopy, to correlate the nitrile stretching frequency with the local electric field exploiting the vibrational Stark effect (VSE). Using MBN SAMs in different metal/SAM interfaces, we sorted out the main factors controlling the nitrile stretching frequency, which comprise, in addition to external electric fields, the metal-MBN bond, the surface potential, and hydrogen bond interactions. On the basis of the linear relationships between the nitrile stretching and the electrode potential, an electrostatic description of the interfacial potential distribution is presented that allows for determining the electric field strengths on the SAM surface, as well as the effective potential of zero-charge of the SAM-coated metal. Comparing this latter quantity with calculated values derived from literature data, we note a very good agreement for Au/MBN but distinct deviations for Ag/MBN which may reflect either the approximations and simplifications of the model or the uncertainty in reported structural parameters for Ag/MBN. The present electrostatic model consistently explains the electric field strengths for MBN SAMs on Ag and Au as well as for thiophenol and mercaptohexanoic acid SAMs with MBN incorporated as a VSE reporter.

## 1. Introduction

Self-assembled monolayers (SAMs) are widely used in various fields of interfacial and surface science ranging from biosensing and biocatalysis to nano- and microelectronics [1–3]. The outstanding importance of metal/SAM devices has motivated a large number of experimental and theoretical studies of their structural, mechanical, and electronic properties. However, a comprehensive understanding of the interfacial potential distribution in metal/SAM building blocks, particularly in electrochemical environments, has not yet been achieved. One reason for this knowledge gap is the lack of accurate experimental data, which in turn requires appropriate and sufficiently sensitive techniques. In most of the studies, electrochemical methods such as cyclic voltammetry (CV) or electrochemical impedance spectroscopy (EIS) have been employed [4,5], providing information about the charge density at the SAM/solution interface as well as the resistance and the capacity of the monolayer. An alternative and promising approach has been introduced by Oklejas *et al.* [6,7] who exploited the vibrational Stark effect (VSE) of the nitrile function, attached to amphiphiles that constitute a SAM on an electrode surface. The VSE is defined as the perturbation of vibrational transitions, here the C≡N stretching mode, by an external electric field. Using surface enhanced Raman (SER) spectroscopy, it was possible to probe the C≡N stretching frequency of the SAM head groups, thereby providing data that contained information about the interfacial electric field.

**Figure 1:**
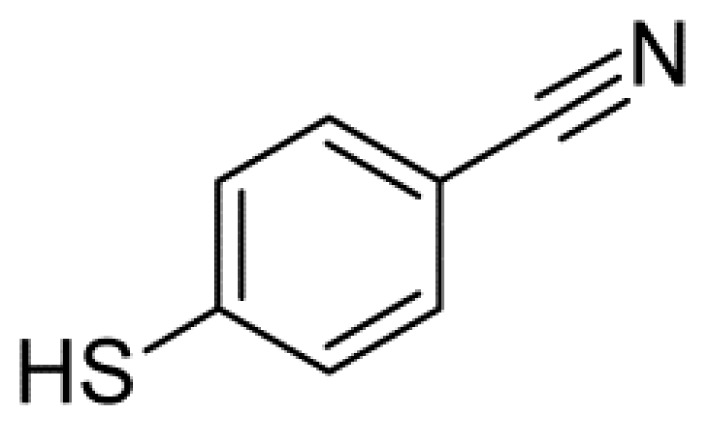
Structural formula of 4-mercaptobenzonitrile (MBN).

A particularly important VSE reporter is 4-mercaptobenzonitrile (MBN; Figure 1), which along with other aromatic nitriles, displays a pronounced sensitivity to the local electric field [8]. Besides its application as a surface modifier, MBN, as well as related molecules, have gained increasing interest as spectral reporters for electric fields in proteins [8–11]. However, regardless of any specific application, it is important to disentangle the various factors controlling the C≡N stretching frequency, which not only depends on local electric fields but also on the particular environment to which the nitrile is exposed. Particularly, hydrogen bonding interactions have been shown to cause frequency shifts even larger than those induced by changes to the local electric field [9,12,13]. Consequently, a profound analysis of the factors influencing the C≡N stretching mode is a prerequisite for correlating frequency shifts with changes of the local electric field, both at electrode/electrolyte interfaces and on protein surfaces.

In an electrochemical environment, the analysis of the VSE represents a particular challenge inasmuch as the electric field strength critically depends on interfacial charge distributions, which in turn are related to the potential of zero charge (E_pzc_). This quantity can readily be determined for bare metal surfaces, but is difficult to assess for SAM-coated metals in aqueous solutions although some attempts have been made [5].

The present study aims at a comprehensive analysis of the VSE of MBN SAMs on Au and Ag electrodes. We have employed surface enhanced infrared absorption (SEIRA) and SER spectroscopy which are the most sensitive techniques for detecting subtle frequency shifts of molecules adsorbed on Au and Ag surfaces, respectively. Probing the C≡N stretching as a function of SAM composition, solvent, and electrode potential, allowed us to disentangle the various factors affecting the nitrile stretching frequency. The results obtained from these experiments constitute the basis for a description of the potential distribution across the electrode/SAM/electrolyte interfaces, thereby refining previous electrostatic models [4,14,15]. It is shown that the analysis of VSE can provide information about important electrostatic properties of metal/SAM interfaces such as their potential of zero charge.

## 2. Results and Discussion

### 2.1. The Nitrile Stretching Modes of MBN in SAM Coated Ag and Au Surfaces

**Figure 2:**
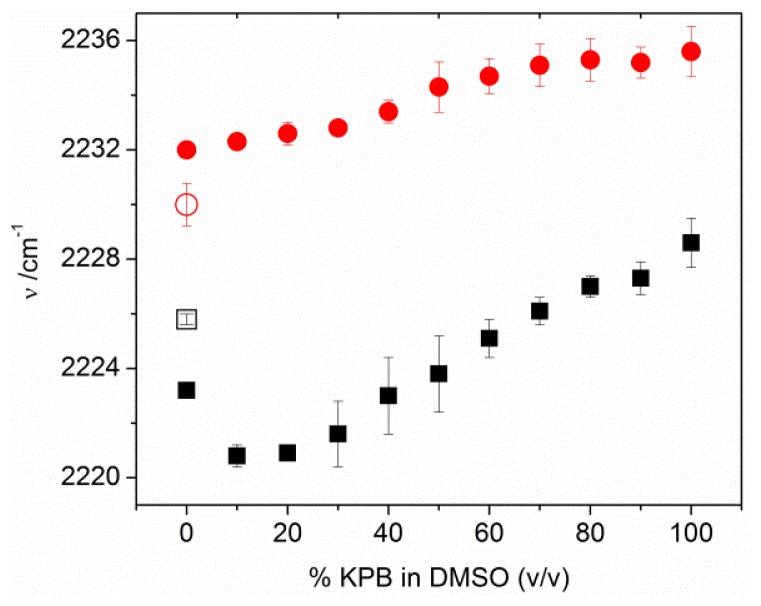
C≡N stretching frequency for MBN self-assembled monolayers (SAMs) on Au (black squares) and Ag (red circles), measured by surface enhanced infrared absorption (SEIRA) and surface enhanced Raman (SER) spectroscopy, respectively. The hollow symbols refer to the measurements of the metal/MBN/air interface whereas the solid symbols indicate the data obtained for metal/SAM interfaces in contact with DMSO/buffer solutions of different composition. The aqueous buffer (KPB) consisted of 10 mM potassium phosphate at pH = 7.0. The error bars indicate the root-mean square deviations over two measurements (see text for further details).

The nitrile stretching frequency of pure MBN SAMs on Ag and Au electrodes, both roughened on the 100-nm scale (see experimental section) was measured by SER and SEIRA spectroscopy, respectively. In the absence of any solvent, *i.e.*, for the SAM/air interfaces, the C≡N stretching frequency was determined to be 2230.0 ± 1.0 and 2225.8 ± 0.2 cm^−1^ for Ag/MBN and Au/MBN, respectively (Figure 2). In the case of Ag/SAM, the frequency increases upon addition of DMSO/buffer solutions with increasing amounts of aqueous buffer (from 0 to 100%) up to a value of 2235.1 ± 1.0 cm^−1^ at 100% aqueous buffer. For Au/SAM in contact with DMSO/buffer solutions, the C≡N stretching frequency first drops considerably (from 0% to 10% buffer) and then steadily increase up to a value 2228.6 ± 1.0 cm^−1^ in 100% buffer solution. This value is essentially the same as that determined also for MBN SAM on Au in 100% water (2228.4 ± 1 cm^−1^).

### 2.2. Potential-Dependent Measurements of MBN-SAMs on Ag and Au Surfaces

**Figure 3:**
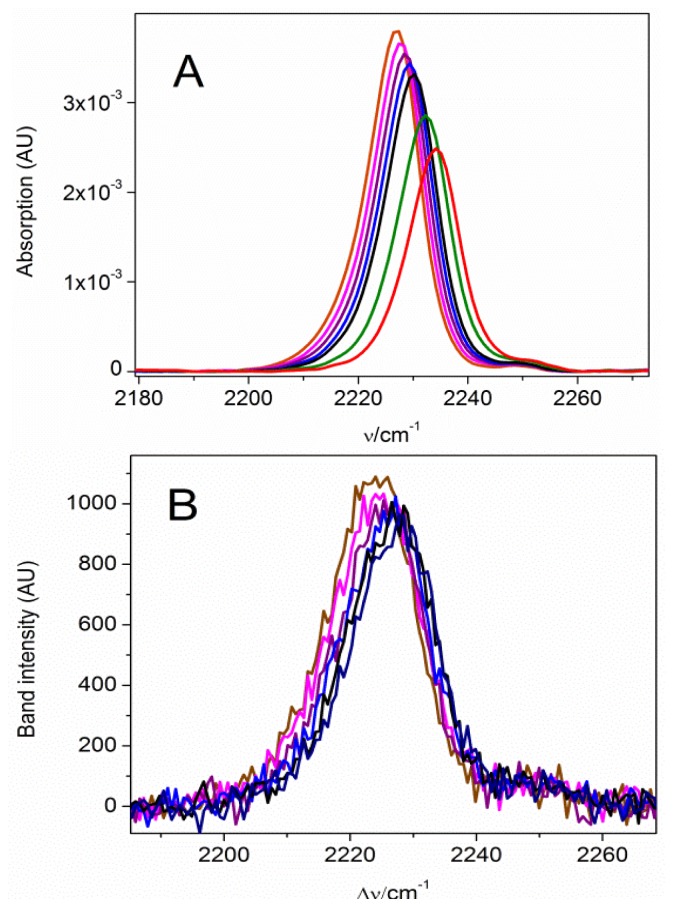
(**A**) SEIRA spectra of Au/MBN and (**B**) SER spectra of Ag/MBN, measured at different electrode potentials *E*. Color code: brown: −0.4 V; pink: −0.3 V; purple: −0.2 V; blue: −0.1 V; black: 0 V; green: +0.3 V; and red: +0.6 V.

**Figure 4:**
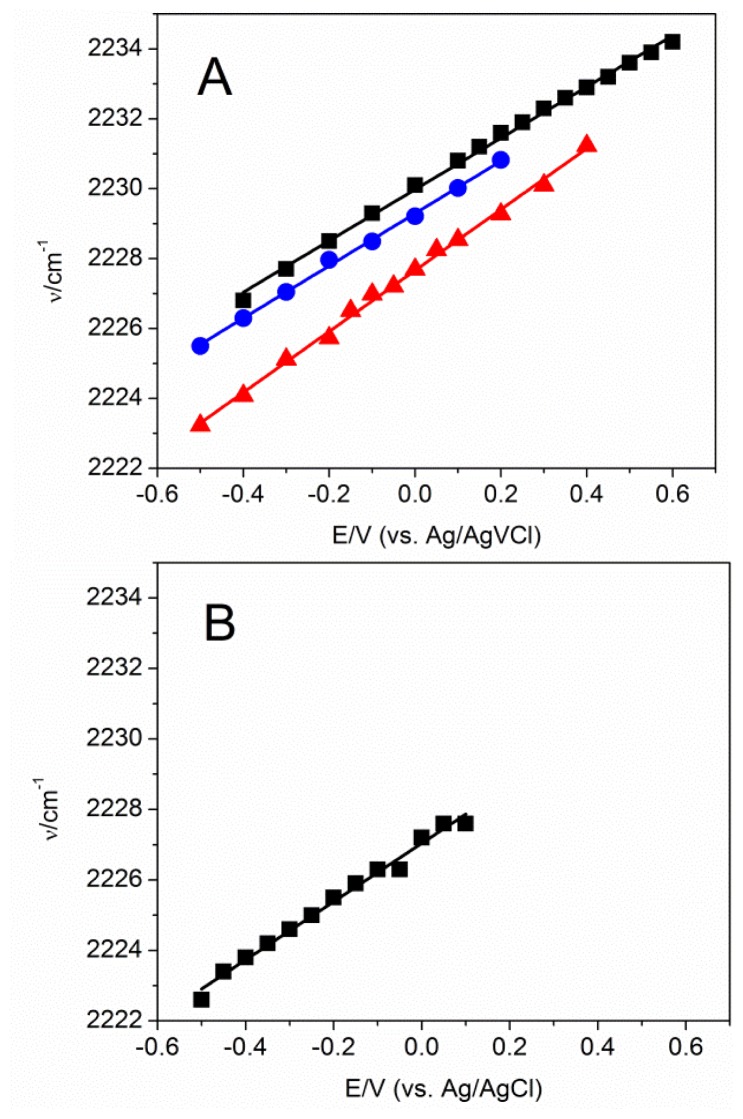
Plots of the nitrile stretching frequency for MBN SAMs on (**A**) Au and (**B**) Ag at different electrode potentials measured by SEIRA and SER spectroscopy, respectively. The black squares in (**A**) and (**B**) refer to the pure MBN SAMs, whereas the blue circles and red triangles in (**A**) represent the data for mixed MBN/thiophenol and MBN/mercaptohexanoic acid (MHA) SAMs on Au, respectively. The solid lines are linear fits to the data including the following slopes *m* and intercepts *b*: Au/MBN: *m* = 8.0 cm^−1^/V^−1^, *b* = 2230 cm^−1^; Au/thiophenol/MBN: *m* = 7.5 cm^−1^/V^−1^, *b* = 2229 cm^−1^; Au/MHA/MBN: *m* = 8.7 cm^−1^/V^−1^, *b* = 2228 cm^−1^; Ag/MBN: *m* = 8.3 cm^−1^/V^−1^, *b* = 2227 cm^−1^.

**Figure 5:**
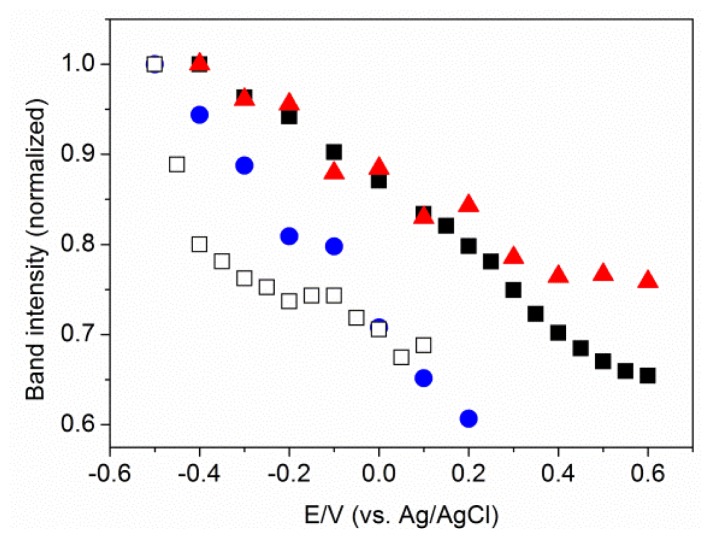
Changes in C≡N stretching band intensities with the electrode potential for Au/MBN (solid black squares), Au/thiophenol/MBN (solid blue circles), and Au/MHA/MBN (solid red triangles) obtained by SEIRA measurements, and Ag/MBN (hollow squares) obtained by SER measurements.

Formation of a MBN SAM on Au and Ag electrode surfaces within the electrochemical cell was monitored by CV (Supplementary Information). The nitrile stretching mode of MBN was probed as a function of the electrode potential, which was varied from −0.4 to +0.6 V and from −0.5 to +0.1 V for Au/SAM and Ag/SAM, respectively. The potential limits were defined by the onset of metal oxidation and reduction of the metal-sulfur bond. A selection of SEIRA and SER spectra is shown in Figure 3. The frequency linearly increases with increasing potential, both for the Au and the Ag electrode. The slope and intercept of the linear fits to the experimental data are similar for the MBN SAMs on Au and Ag (Figure 4). There is no indication of potential-dependent variations in the band profiles. However, in both cases, peak intensity decreases significantly as the potential becomes more positive (Figure 5). This potential-dependent intensity variation has been found to be reversible.

### 2.3. MBN in Mixed SAMs on Au Electrodes

**Figure 6:**
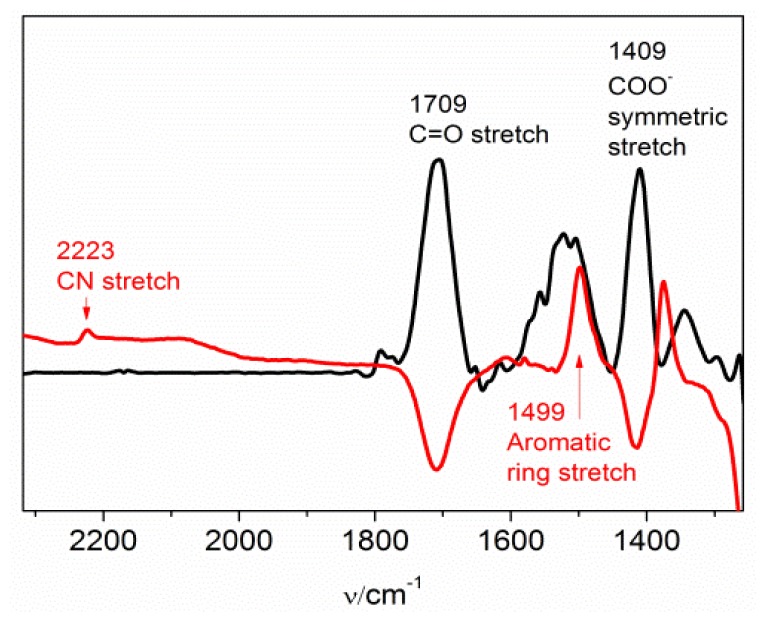
SEIRA spectrum of the MHA SAM on Au (**A**, black trace), measured at open circuit, compared with the SEIRA spectrum obtained after addition of MBN to the solution at an electrode potential of −0.4 V (**B**, red trace), using spectrum **A** as a reference. The negative bands in spectrum **B** refer to the removal of MHA from the surface, whereas the positive bands indicate the incorporation of MBN into the SAM.

To probe the behavior of the MBN nitrile stretching mode within an environment of charged residues, SEIRA-active Au electrodes were coated with SAMs of mercaptohexanoic acid (MHA). Subsequently, MBN was added to the solution under controlled potential. To integrate MBN into a MHA monolayer, a potential of −0.4 V was applied to remove a fraction of MHA molecules from the surface such that binding of MBN from the bulk solution to the vacant adsorption sites became possible. The process was monitored by SEIRA spectroscopy on the basis of the decreasing band intensities for the carboxylate stretching modes of MHA and increasing intensity of the nitrile stretching of MBN (Figure 6). After ca. 12 hours, the SEIRA intensity of the nitrile stretching has reached a value of ca. 4% of that for the pure MBN monolayer at the same potential, reflecting the relative contribution of MBN to the composition of the mixed monolayer. Subsequently, potential-dependent SEIRA measurements were carried out to probe the nitrile stretching frequency of MBN within an environment dominated by MHA with protonated and deprotonated head groups. Similar to the pure MBN, the frequency follows a linear relationship with the electrode potential, albeit with slightly different slope and intercept (Figure 4). The larger slope, compared to Au/MBN, may imply that the nitrile groups are less exposed to the aqueous phase [16] and are instead partly buried within the SAM.

Incorporation of MBN into a thiophenol SAM on an Au electrode was achieved in a similar way, however, at an electrode potential of 0.0 V. The nitrile stretching was detected by SEIRA spectroscopy. This band remains after removal of excess MBN from the cell and careful rinsing the electrode (Supplementary Information). Detachment of MBN from the electrode was only possible by reductive desorption, *i.e.*, upon applying an electrode potential of −1.5 V that led to an irreversible loss of the band. Potential-dependent SEIRA measurements demonstrated again a behavior similar to that of a pure MBN SAM (Figure 4). In this case, however, the slope is lower than for the pure SAM, possibly indicating that in this system the C≡N groups are more exposed to the solvent [16].

### 2.4. Factors Controlling the Zero-Field Nitrile Stretching Frequency

For the analysis of the VSE of MBN we use the linear dependence of the C≡N stretching frequency ν on the electric field $\vec{E_{F}}$ according to Equation (1):

(1) $$\nu =\nu_{0}-\Delta \vec{\mu }\cdot \vec{E_{F}}$$

where *Δμ⃗* is the difference dipole moment (between the ground and excited vibrational states) and ν_0_ is the frequency in the absence of an external electric field. Neglecting the higher terms in the electric field dependence is justified *a posteriori* (*vide infra*). For MBN and other nitrile-containing VSE labels, *Δμ⃗* has been determined both in frozen 2-methyl-tetrahydrofurane glass and after attachment to a protein [8,16], indicating that this quantity is lowered by about 20% in aqueous solutions compared to a hydrophobic environment [16]. Accordingly, we have scaled the value of *Δμ⃗* = 7.4 × 10^−9^ cm^−1^·V^−1^·m, determined for MBN in the hydrophobic solvent 2-methyl-tetrahydrofurane [8], by a factor of 0.8 to obtain an approximate value for MBN SAMs with the nitrile function exposed to the aqueous solution, *i.e.*, *Δμ⃗* = 6.0 × 10^−9^ cm^−1^·V^−1^·m. The value for the zero-field frequency ν_0_ determined for a protein-attached MBN in an aqueous environment, as well as that determined for MBN in a frozen 2-methyl-tetrahydrofurane glass cannot be adopted for MBN SAMs, as electric field and hydrogen bonding interactions, as well as binding to the different metals and the surface potential of the SAM may affect the C≡N stretching frequency.

The Au/MBN/air and Ag/MBN/air systems present C≡N stretching frequencies at 2225.8 and 2230.0 cm^−1^, respectively. For comparison, the C≡N stretching frequency of benzonitrile in the gas phase is observed at 2238 cm^−1^ [17], indicating a decrease in the C≡N stretching frequency upon metal binding and SAM formation.

Upon inserting the MBN-coated metal electrodes into a DMSO solution, the polar solvent molecules tend to orient in the SAM/solvent interface. The resulting surface potential causes a downshift of the C≡N stretching frequency by 2.6 cm^−1^ in the case of Au. Taking into account the potential-dependence of the C≡N stretching frequency (Figure 4), a frequency downshift is equivalent to a negative shift of the electrode potential. Accordingly, we conclude that the DMSO molecules are preferentially aligned with the partially negatively charged oxygen atoms facing the SAM surface. Conversely, a 2-cm^−1^ upshift is observed in the case of Ag/MBN in contact with 100% DMSO, pointing to the opposite orientation of the DMSO molecules with respect to the SAM surface.

In addition to the above observations, the response of the C≡N stretching frequency to the admixture of aqueous buffer is different for Au/MBN and Ag/MBN. The further decrease of the frequency at small amounts of buffer for Au/MBN may be attributed to the parallel orientation of DMSO and the even more polar water molecules, both with the oxygen atoms pointing to the SAM, such that surface potential becomes more negative. However, with increasing buffer content, hydrogen bonding interactions of water molecules with the nitrile group gain importance. Such interactions have been shown to exert the opposite effect on the C≡N stretching frequency [9,12,13] which eventually dominates and thus leads to an increase of the frequency with the buffer content. In contrast, in the Ag-bound MBN SAM the nitrile stretching frequency increases monotonically, since in this case the solvent molecule (DMSO, H_2_O) orientation and hydrogen bonding affect the nitrile stretching in the same direction. It is interesting to note that the C≡N stretching frequency of MBN in DMSO solution was recorded at 2227.7 cm^−1^ and the corresponding value for an aqueous solution was estimated to be 2233.6 cm^−1^ (Supplementary Information). These values lie between the respective frequencies for the Au- and Ag-bound MBN SAM, consistent with the conclusion that the SAM-coated metal surfaces are oppositely charged at open circuit.

On the basis of these results we may now estimate the zero-field frequency ν_0_ of the Ag/SAM/buffer and Au/SAM/buffer systems, *i.e.*, the frequency that includes the effect of metal-sulfur bond formation and the hydrogen bonding interaction in the aqueous solution while excluding the effect of the (open circuit) metal electrode potential and thus that of the surface potential at the SAM/solution interface. This value is experimentally not accessible. However, we may assume that the effect of hydrogen bonding on the C≡N stretching frequency in an aqueous environment is independent of the existence of the metal-sulfur bond. Correspondingly, the frequency upshift of 7.6 cm^−1^ (*Δν*_HB_) from solid MBN (2226 cm^−1^ [11]) to MBN in aqueous solution (2233.6 cm^−1^) (Supplementary Information) should be approximately the same as the frequency shift arising from the transfer of the Ag/MBN/air (2230.0 cm^−1^) and Au/MBN/air (2225.8 cm^−1^) systems into an aqueous solution in the absence of any effects due to the electrode charge. Correspondingly, the addition of the *Δ*ν_HB_ increment of 7.6 cm^−1^ to the frequencies of the metal/MBN/air interfaces is considered to be a reasonable approximation for the zero-field frequencies and we thus obtain ν_0_ = 2237.6 cm^−1^ and ν_0_ = 2233.4 cm^−1^ for the MBN SAMs on Ag and Au, respectively.

### 2.5. Electrostatic Model

On the basis of an electrostatic description of the interfacial potential distribution for SAM-coated devices by Smith and White [4] and its further developments [14,15], the electric field at the SAM/solution interface is given by Equation (2):

(2) $$|\vec{E_{F}}|=\frac{{ɛ}_{S}\kappa {ɛ}_{0}}{{ɛ}_{0}({ɛ}_{C}+{ɛ}_{S}\kappa d_{C})}\left(E-E_{pzc}\right)-\frac{\sigma_{C}}{{ɛ}_{0}({ɛ}_{C}+{ɛ}_{S}\kappa d_{C})}$$

where ɛ_c_ and ɛ_s_ are the dielectric constants of the SAM and aqueous solution, respectively; d_c_ is the thickness of the MBN monolayer, ɛ_0_ denotes the permittivity, and κ is the reciprocal Debye length. The term σ_c_ refers to the charge density on the SAM surface which for carboxyl-terminated SAMs has been related to apparent pK_a_ of the head groups. In a more general sense, σ_c_ may be regarded as the charge density in the inner Helmholtz layer reflecting the contributions of charged SAM head groups as well as those of the ions and water dipoles aligned in the immediate vicinity of the SAM surface. This surface charge density, originally assumed to be constant, varies with the electrode potential E, or more precisely, with the difference between the electrode potential E and the potential of zero charge of the metal E_pzc_. Here, we assume in a first approximation a linear relationship according to Equation (3):

(3) $$\sigma_{C}=\sigma_{0}+k\left(E-E_{pzc}\right)$$

where *k* is a constant and σ_0_ is the charge density at *E*_pzc_. Equation (3) may be combined with Equation (2) to afford Equation (4)

(4) $$|\vec{E_{F}}|=\frac{{ɛ}_{S}\kappa {ɛ}_{0}}{{ɛ}_{0}({ɛ}_{C}+{ɛ}_{S}\kappa d_{C})}\left(E-E_{pzc}\right)-\frac{\sigma_{0}}{{ɛ}_{0}({ɛ}_{C}+{ɛ}_{S}\kappa d_{C})}-\frac{k\left(E-E_{pzc}\right)}{{ɛ}_{0}({ɛ}_{C}+{ɛ}_{S}\kappa d_{C})}$$

and after rearrangement:

(5) $$|\vec{E_{F}}|=\frac{{ɛ}_{S}\kappa {ɛ}_{0}-k}{{ɛ}_{0}({ɛ}_{C}+{ɛ}_{S}\kappa d_{C})}\left(E-E_{pzc}\right)-\frac{\sigma_{0}}{{ɛ}_{0}({ɛ}_{C}+{ɛ}_{S}\kappa d_{C})}$$

Note that Equation (5) indicates a linear relationship between the electrode potential and the electric field which, taking into account the observed linear correlation between the nitrile stretching frequency and *E* (Figure 4), justifies neglecting higher order terms in the analysis of the VSE (cf. Equation (1)). For the electric-field dependence of the C≡N stretching frequency, we have to take into account the definition of the nitrile dipole axis pointing from the carbon to the nitrogen such that *Δμ⃗* has a negative sign [18]. Thus combining Equation (5) and Equation (1) affords:

(6) $$v=\nu_{0}+|\Delta \vec{\mu }|cos\theta \left(\frac{{ɛ}_{S}\kappa {ɛ}_{0}-k}{{ɛ}_{0}({ɛ}_{C}+{ɛ}_{S}\kappa d_{C})}\left(E-E_{pzc}\right)-\frac{\sigma_{0}}{{ɛ}_{0}({ɛ}_{C}+{ɛ}_{S}\kappa d_{C})}\right)$$

where θ refers to the angle formed by the vector of the nitrile dipole moment and that of the electric field, arising from the scalar multiplication (see Equation (1)). Rearranging Equation (6) leads to:

(7) $$v=\nu_{0}-|\Delta \vec{\mu }|cos\theta \left(\frac{({ɛ}_{S}\kappa {ɛ}_{0}-k)E_{pzc}+\sigma_{0}}{{ɛ}_{0}({ɛ}_{C}+{ɛ}_{S}\kappa d_{C})}\right)+|\Delta \vec{\mu }|cos\theta \left(\frac{{ɛ}_{S}\kappa {ɛ}_{0}-k}{{ɛ}_{0}({ɛ}_{C}+{ɛ}_{S}\kappa d_{C})}\right)E$$

where the first two terms on the right hand side of Equation (7) account for the intercept (*b*) of the linear fits in Figure 4, and the last term corresponds to the slope (*m*). From the slope term one may determine the quantity k for the various metal/SAM interfaces (Table 1).

For these calculations, estimates for the tilt angle of the nitrile group are required. For MBN on Au θ was determined to be ~40° [24], which is similar to that for a thiophenol monolayer on Au (49°–54°) [25,26]. Reported angles for MBN and thiophenol on Ag, however, differ significantly, *i.e.*, 24°–28° for thiophenol on Ag [27,28], and, most surprisingly, ca. 0° for MBN [29]. In view of this striking discrepancy between thiophenol and MBN on Ag, which is in sharp contrast to the findings for Au, one has to consider the reported value for Ag/MBN with caution. Nevertheless, for the present calculations we first adopt 49° and 0° for Au/MBN and Ag/MBN, respectively. These values are also included in determining the thickness of the SAM, d_c_, using an Ag-S bond length of 2.4 Å and Au-S bond length of 1.9 Å [30,31], a sulfur-nitrogen distance of 6.5 Å as well as the respective values of θ discussed above. As a result, d_c_ is evaluated to be 6.8 × 10^−10^ m and 8.9 × 10^−10^ m for Au/MBN and Ag/MBN, respectively.

Now we consider the potential at which the electric field at the position of the nitrile group is zero (Equation (5)). This potential may be considered as the effective potential of zero-charge E_0_ for the entire metal/SAM/solution system. Expressing Equation (7) for *E* = *E*_0_, in terms of the slope m and intercept b of the linear fits to the experimental data in Figure 4, we then obtain

(8) $$E_{0}=\frac{\nu_{0}-b}{m}$$

Note that *E*_0_ may, therefore, differ substantially from *E*_pzc_, which refers to the potential of zero-charge of the bare metal. These experimentally determined *E*_0_ values are listed in Table 1. We further use these *E*_0_ values obtained in this way to calculate the charge densities in the inner Helmholtz layer at zero-field (σ_0_) according to Equation (9):

(9) $$\left(E_{0}-E_{pzc}\right)({ɛ}_{S}\kappa {ɛ}_{0}-k)=\sigma_{0}$$

which is derived from Equation (7) when E = E_0_ and ν = ν_0_.

**Figure 7:**
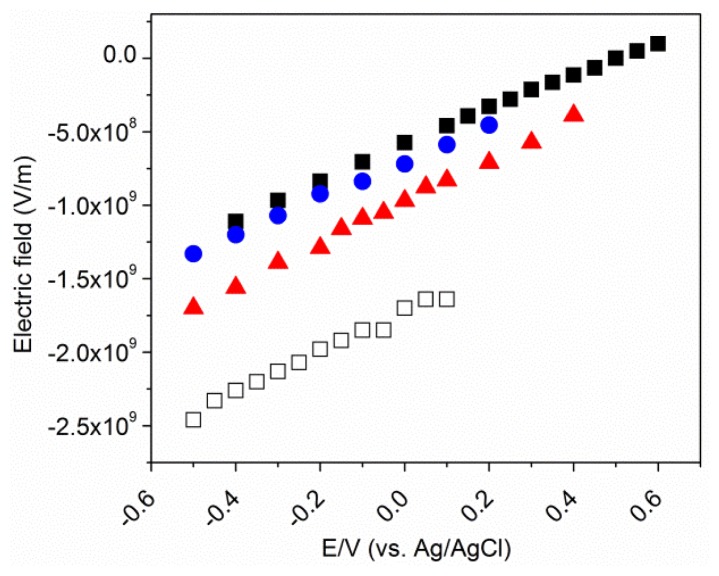
Plots of the electric field strength as a function of the electrode potential, calculated according to Equations (1) and (5), using the experimentally determined C≡N stretching frequencies for Au/MBN (solid black squares), Au/thiophenol/MBN (solid blue circles), Au/MHA/MBN (solid red triangles), and Ag/MBN (hollow squares).

Equation (5) may now be used to calculate the variation of the electric field strength at the SAM surface, *i.e.*, at the position of the nitrile group, as a function of the electrode potential (Figure 7). For all metal/SAM devices, the modulus of the field strength increases with decreasing potential, covering a range from 0.9 × 10^8^ V/m to −2.5 × 10^9^ V/m. Whereas similar values are obtained for Au/MBN and Au/thiophenol/MBN, the modulus of the field strength is larger in the mixed MHA/MBN SAM on Au.

As judged from the SEIRA intensity, this mixed SAM includes only 4% MBN, however, it is not known if the incorporation of MBN into a preformed MHA SAM leads to a largely homogeneous MBN distribution or to MBN islands within the MHA SAM. In the latter case, one would expect an inhomogeneity of the SEIRA bands, given that there are different electric field strengths at the surface of the dominant MHA part of the SAM and the much smaller MBN islands, leading to different nitrile stretching frequencies in the interior of the MBN island compared to the MBN/MHA boundary. Such inhomogeneities are not observed in the SEIRA spectra of Au/MHA/MBN, implying that the electric field at the SAM surface does not display significant gradients parallel to the surface or that the distribution of MBN within the MHA SAM is largely homogeneous. In any case, we may conclude that (i) MBN probes the electric field on the MHA SAM surface, and (ii) the magnitude of the electric field on a MHA SAM on Au at pH 7.0 is only larger by ca. 15% compared to Au/MBN, despite the partial dissociation of the carboxylate head groups at pH 7.0 [5,14,32,33]. This conclusion is consistent with the finding that the magnitude of the electric field on Ag/MBN, which is ca. twice as large as that found for Au/MBN, is quite similar to that estimated for Ag/MHA monolayers [14].

### 2.6. The Effective Potential of Zero Charge

The quantity *E*_0_ derived from the experiments reflects the change in the potential of zero charge of the pure metal (*E*_pzc_) due to binding of the dipolar SAM to the metal surface. The effective potential of zero charge *E*_0_ can also be estimated by calculating the change in the work function of the metal (ΔΦ) due to the coating by the SAM. In one approach, suggested by Wang *et al.* [22], the only parameters affecting Δ*Φ* are assumed to be the direction of the dipole moment of the SAM and its angle with respect to the surface normal (*β*), which can be evaluated from the tilt angle θ for the nitrile function, taken to be 49° and 0° for Au/MBN and Ag/MBN, respectively (*vide supra*). On the basis of the Δ*Φ vs.* cos(*β*) relationship determined by Wang *et al.* [22], one then obtains Δ*Φ* = 0.15 and 2.6 eV for Au/MBN and Ag/MBN respectively.

For bare metals, the work function Φ_M_ is related to the potential of zero charge according to:

(10) $$E_{pzc}=\Phi_{M}-K$$

where here E_pzc_ is expressed in V *vs.* NHE and *K* is a constant that was determined to be 5.01 and 4.61 eV for Ag and Au, respectively [34,20]. Recommended values for the work function of bare polycrystalline Ag and Au are 4.3 and 4.88 eV [21,35] such that one obtains −0.71 V and +0.27 V for *E*_pzc_ of polycrystalline Ag and Au, respectively. Correspondingly, Equation (10) allows evaluating the effective potential of zero charge from the work function of the SAM-coated metals *Φ*_M/SAM_ according to Equation (11):

(11) $$E_{0}=\Phi_{M/SAM}-K=\Phi_{M}+\Delta \Phi -K$$

such that one obtains, after translating the reference potential scale from NHE to Ag/AgCl, E_0_ = 0.215 and 1.658 V for Au/MBN and Ag/MBN, respectively. The values are in reasonable agreement with the data derived from the VSE analysis (Table 1), specifically in view of the underlying approximations of both the electrostatic model and the E_0_ calculations.

An alternative for calculating Δ*Φ*, suggested by Heimel *et al.* [23,36], includes the metal-sulfur bond dipole, BD, and the potential difference across the dipolar SAM, Δ*V*_vac_, according to Equation (12):

(12) $$\Delta \Phi =\Delta V_{vac}+BD$$

here ΔV_vac_ is given by Equation (13):

(13) $$\Delta V_{vac}=-\frac{\mu_{⊥}}{{ɛ}_{0}{ɛ}_{eff}A}$$

where *μ*_⊥_ is the dipole moment component of the SAM in the direction of the surface normal, *A* is the area of the unit cell, and *ε_eff_* is a correction coefficient suggested to be 2.3 for a SAM of 4-mercaptobiphenylnitrile [23]. Estimating *μ*_⊥_, even if *β* is known, is not straightforward as it differs from the mere projection of the single MBN molecular dipole onto the surface normal, due to the depolarization of the MBN dipole by neighboring molecules in the SAM [37]. According to Natan *et al.* [37], we adopt a value of 3.6 Debye for the dipole moment of the MBN SAM, which is then multiplied by cos(*β*) to afford *μ*_⊥_. On the basis of calculated values for a series of related systems [23], BD is taken to be −1.05 eV and −0.27 eV for Au/MBN and Ag/MBN, respectively. The difference between these two values is in good agreement with experimental data reported by Alloway *et al.* [38]. Thus, one obtains 0.3 eV (Au/MBN) and 2.9 eV (Ag/MBN) for Δ*Φ* and eventually *E*_0_ = 0.402 and 1.936 V *vs.* Ag/AgCl for Au/MBN and Ag/MBN, respectively. Here, *E*_0_ for Au/MBN is much closer to the experimentally derived value (Table 1) than in the previous approach, while there is a stronger deviation from E_0_ for Ag/MBN. The failure to reproduce the experimentally derived value for Ag/MBN in a satisfactory manner does not necessarily argue against the approach proposed by Heimel *et al.* [23]. In view of the very good agreement for Au/MBN, one may also question the most uncertain parameter in the calculation for Ag/MBN, *i.e.*, the tilt angle of the nitrile function (θ) and thus the angle of the dipole moment of the entire molecule with respect to the surface normal (β). One may, therefore, take the approach suggested by Ballav *et al.* [16] to estimate the angles β and θ starting with *E*_0_ = 1.277 V, *i.e.*, the experimentally derived value. Under these conditions, a β angle of 38° is predicted, corresponding to a tilt angle (θ) of 26°, which is in good agreement with those reported for thiophenol on Ag [27,28].

Either way, it should be noted that previous experimental and theoretical studies on SAM structures and specifically on the tilt angle refer to the SAMs on smooth metals in air or *in vacuo*. Possible structural differences of the SAMs due to the nanostructured metal surfaces or the contact with an aqueous solution or in an electrochemical environment are not considered. Moreover, the present analysis tacitly assumes that the tilt angle of the nitrile function is potential-independent. The variation in the SEIRA peak intensities in Figure 5 allows one to estimate the underlying error of this assumption. In the ATR SEIRA set-up, the IR signals are enhanced via the electric field component of the electromagnetic radiation perpendicular to the surface |E⃗*_rad_*|_⊥_, such that the maximum enhancement is achieved for molecular oscillators that are oriented in that direction. Since the SEIRA intensity I_SEIRA_ scales with the square of |E⃗*_rad_*|_⊥_, it follows that I*_SEIRA_* ∝ (cosθ)^2^. Assuming that the SEIRA intensity for a pure MBN SAM on an Au electrode, measured at an electrode potential of +0.1 V, would refer to a tilt angle of the nitrile function with respect to the surface normal of 49° (*vide supra*) the increased and decreased intensities at −0.4 V and +0.6 V then corresponded to a tilt angle of 45° and 57°, respectively.

## 3. Experimental Section

### 3.1. Materials

6-Mercaptohexanoic acid (MHA), 4-mercaptobenzonitrile (MBN), and thiophenol were purchased from Dojindo, Apin Chemicals, and Sigma-Aldrich, respectively. All other chemicals and solvents were of the highest purity grade available. Water was purified by a Millipore system and had a resistivity > 18 MΩ·cm.

### 3.2. SAM Preparation

Prior to SAM deposition, Au films and Ag ring electrodes were prepared and electrochemically roughened as described previously [39,40]. MBN SAMs on Ag were obtained by immersing the electrode in a solution of 1 mM MBN in dimethyl sulfoxide (DMSO):H_2_O (3:1 v/v) at 4 °C for 18 h; for SAM formation on the Au surface, a 1 mM MBN solution in anhydrous DMSO was employed. Mixed thiophenol/MBN SAMs on Au were prepared in two steps. First, a pure thiophenol SAM was formed from a 2 mM solution of thiophenol in DMSO:H_2_O 3:1 (v/v), using the same procedure as for MBN SAM formation in the spectro-electrochemical cell. Subsequently, the cell was washed successively with DMSO, ethanol, and water. Afterwards a 10 mM phosphate buffer solution (pH = 7) was added to the cell. Finally, the addition of MBN dissolved in DMSO:H_2_O 3:1 (v/v) to the cell under potential control (0 V *vs.* Ag/AgCl) resulted in a final concentration of 100 nM. In a similar way, a mixed MHA/MBN SAM was formed, starting with the pure MHA SAM [11,39] followed by addition of MBN (10 μM) to the solution at −0.4 V. The formation of the SAMs was monitored by CV and SEIRA spectroscopy (Supplementary Information).

### 3.3. Spectro-Electrochemical Measurements

SEIRA measurements were carried out in the Kretschmann-ATR configuration which was integrated in an electrochemical cell for potential-controlled studies. Details of the set-up are given elsewhere [39]. SEIRA spectra were recorded with a spectral resolution of 4 cm^−1^ on a Bruker IFS66v/s spectrometer, using a liquid nitrogen cooled photoconductive MCT detector. 400 scans were co-added for each spectrum; three to five spectra were recorded for each experiment and averaged. Each SEIRA experiment was repeated at least twice.

SER spectroelectrochemical experiments were carried out with the set-up described previously [41]. The spectra were obtained with the 413-nm excitation line of a Krypton ion laser using a confocal Raman spectrometer equipped with a liquid nitrogen cooled CCD detector. The spectral resolution was better than 2 cm^−1^. The power of the focused laser beam on the sample was 1 mW. The accumulation time was 60 s. All experiments were repeated four times.

CV measurements were carried out in the same cells used for SEIRA and SER spectroelectrochemistry [39,41]. In all electrochemical and spectroelectrochemical measurements potassium phosphate buffer (10 mM, pH = 7.0) was used. Unless otherwise indicated, all potentials cited in this work refer to the Ag/AgCl electrode (3 M KCl).

## 4. Conclusions

As a prerequisite for exploiting the VSE of MBN monolayers in electrochemical environments, the various factors affecting the stretching frequency of the nitrile function were analyzed and shown to include the effect of (i) MBN binding to the metal and the formation of a SAM, (ii) the surface potential due to the alignment of polar solvent molecules on the SAM surface, (iii) hydrogen bonding interactions in aqueous solutions, and finally (iv) the external electric field. The potential dependence of the C≡N stretching is linear in the potential ranges studied for the various metal/SAM systems, which facilitates the translation of frequency shifts into local electric fields. For this analysis, an electrostatic description is presented that, unlike previous models, takes into account the potential-dependent variation of the charge density in the inner Helmholtz layer of the SAM. For Au/MBN, the effective potential of zero charge derived from this model is in good agreement with the values calculated from literature data. The more significant deviations in the case of Ag/MBN may either be related to the approximations and simplifications in the present model or to the uncertainty in the tilt angle value taken from the literature. The present electric field analysis for mixed MHA/MBN is consistent with previous results for pure MHA, thus indicating that MBN may be used as a reporter group for *in situ* monitoring of local electric field in SAMs that are used for binding biomolecules such as redox proteins and enzymes [11,42,43].

## Supplementary Information



## Figures and Tables

**Table 1:** Electrostatic parameters for various metal/MBN interfaces a.

	Slope b (cm^−1^/V)	Intercept b (cm^−1^)	k c (CV^−1^m^−2^)	E_0_, exp. c (V)	σ_0_ c (Cm^−2^)	E_0_, calc. d (V)	E_0_, calc. e (V)
Ag/MBN	8.3	2227	−0.117	1.277	1.005	1.685	1.936
Au/MBN	8.0	2230	−0.186	0.425	0.199	0.215	0.402
Au/TP f/MBN	7.5	2229	−0.153	0.587	0.267	-	-
Au/MHA/MBN	8.7	2228	−0.232	0.621	0.330	-	-

a calculated according to Equation (7), using ɛ_S_ = 78, *κ* = 5·10^8^ m^−1^, ɛ_0_ = 8.854 × 10^−12^ C·V^−1^·m^−1^, ɛ_c_ = 3 [19], Δμ = 6.0 × 10^−9^ cm^−1^·V^−1^·m, and d_c_ = 6.8 × 10^−10^ m for Au/MBN and 8.9 × 10^−10^ m for Ag/MBN as described in the text. For E_pzc_, we have used −0.92 V and +0.06 V for of polycrystalline Ag and Au, respectively [20,21];

b taken from the linear fits in Figure 4;

c as defined by Equation (3), derived from the experimental data according to Equations (7) and (8);

d calculated using Equation (11), according to Wang *et al.* [22];

e calculated using Equations (11–13), according to Heimel *et al.* [23];

f TP: thiophenol.
