# Supplementary material for: Vibrational Stark Effect of the Electric-Field Reporter 4-Mercaptobenzonitrile as a Tool for Investigating Electrostatics at Electrode/SAM/Solution Interfaces
Source: Int J Mol Sci. 2012 Jun 18;13(6):7466–82. doi: 10.3390/ijms13067466 (PMC3397537; doi:10.3390/ijms13067466)
Supplement: Supplementary file 1 [file ijms-13-07466-s001.pdf]

Vibrational Stark Effect of the Electric-Field Reporter 4-Mercaptobenzonitrile as a Tool for Investigating Electrostatics at Electrode/SAM/Solution Interfaces

Supplementary Information

Content:

1. MBN-SAM on Au electrodes

2. Thiophenol:MBN mixed monolayer

3. MBN-SAM on Ag electrodes

4. The nitrile stretching frequency of MBN in an aqueous solution

1. MBN-SAM on Au Electrodes

Figure S1 (top) shows the increase in nitrile stretching intensity that occurs when a typical MBN SAM is formed on Au. Upon addition of MBN dissolved in DMSO to the SEIRA cell, the CN stretch peak appeared at 2222.8 cm^{-1}. The increase in peak intensity was followed by SEIRA. In Figure S1 (bottom) the time course of the peak intensity is shown. The peak intensity first grows rapidly and then reaches a plateau as the SAM becomes too tightly packed to admit any further MBN molecules at the surface. When the cell is washed with DMSO to remove excess MBN, the CN stretch reaches a frequency of 2223.2 cm^{-1}.

To make sure MBN has indeed formed a monolayer on the gold surface, a cyclic voltammogram (CV) of the monolayer was recorded. Subsequently, after the completion of the potential series, cyclic voltammetry (CV) was performed, with vertex potentials at −400 and 300 mV, and a scan rate of 50 mV/sec, followed by the acquisition of a SEIRA spectrum. Subsequently a potential of −1500 mV was applied for 5 min to desorb the SAM. A SEIRA spectrum was again acquired and a second, identical CV performed. A final SEIRA spectrum was acquired. In Figure S2 (top), the CV measured before applying the reductive potential shows the small capacitive current indicative of the presence of a SAM [1], whereas after applying −1.5 V, the Au-S bond is reduced and the SAM is removed from the gold surface, leading to a sharp increase in the capacitive current, and yielding a CV similar to that obtained for the bare gold surface. In Figure S2 (bottom) the MBN nitrile stretch peak is seen in a SEIRA spectrum recorded before applying the reductive potential, and it disappears as the Au-S bond is reduced and the MBN SAM is removed from the surface.

Figure S1. Time series of the CN stretch vibration arising from MBN monolayer formation on Au film in SEIRA setup. **Top:** SEIRA spectrum segment; **bottom:** Peak height at 2222.8 cm^{-1} as a function of time after addition of MBN to SEIRA cell.

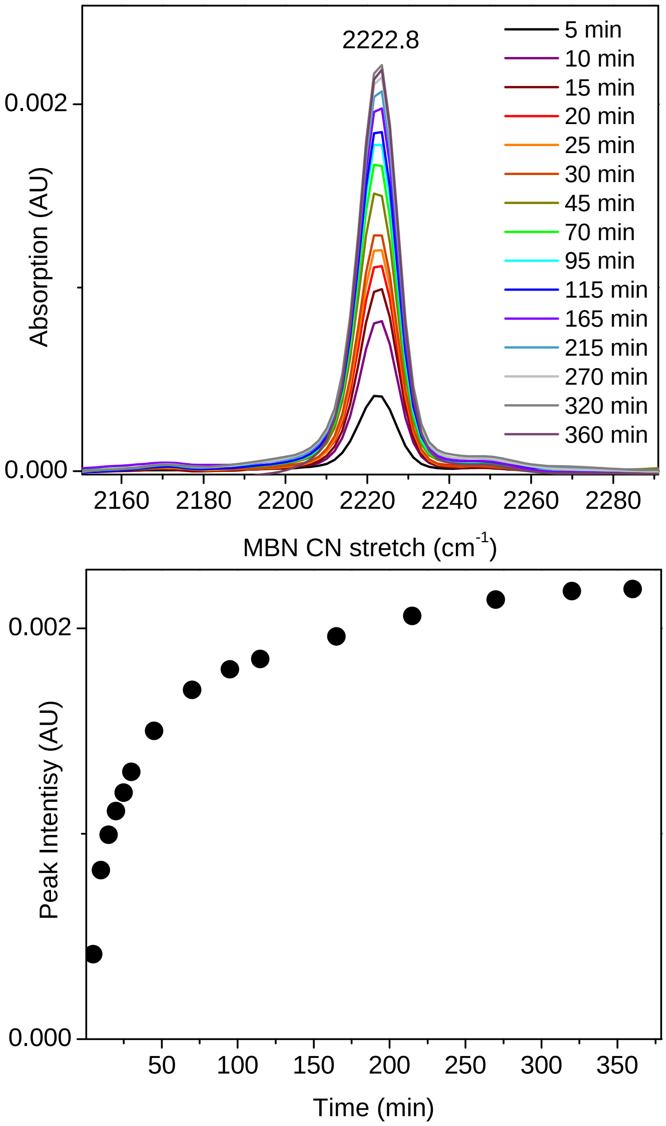

Figure S2. Top: Cyclic voltammograms measured in the SEIRA cell, of the bare gold film (black), of the gold film coated with MBN SAM (blue), and of the gold film after applying a voltage of −1.5 V to the MBN SAM (red). **Bottom:** SEIRA spectra, showing the MBN peak before (black), during (red) and after (blue and green) the application of −1.5 V to the gold film.

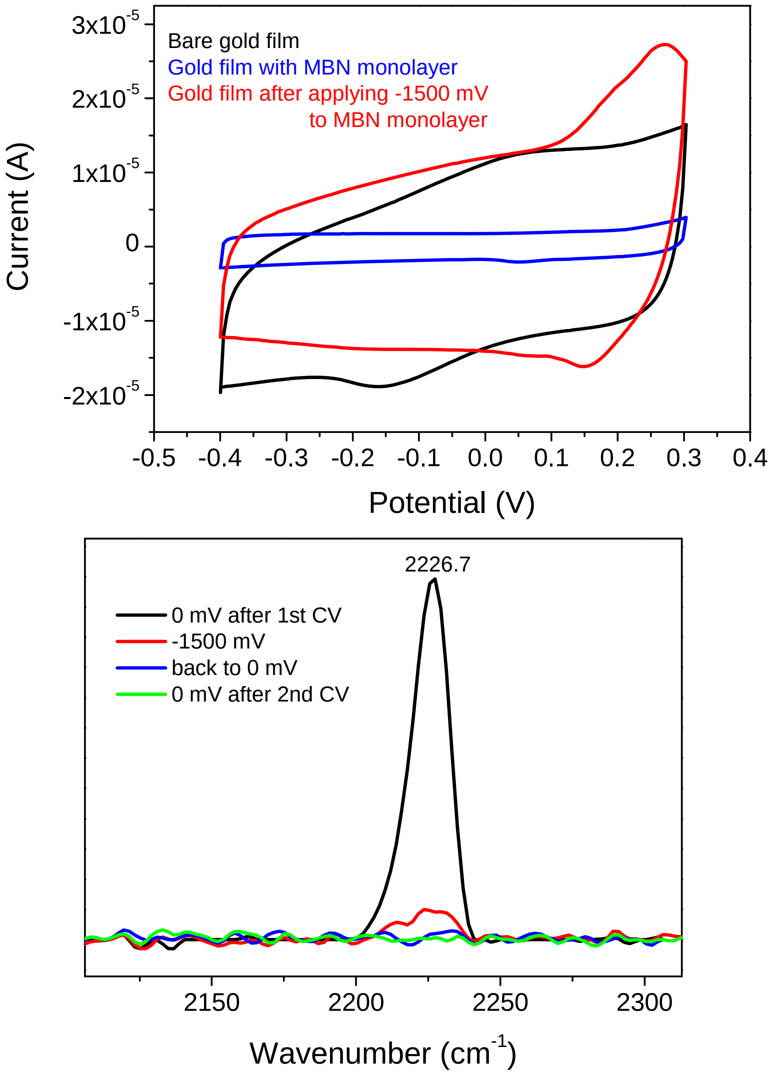

2. Thiophenol:MBN Mixed Monolayer

The SEIRA cell was incubated overnight with a 2mM solution of Thiophenol in DMSO:H_2O 3:1 (v/v). The next day, the solution was removed from the cell, and the cell was successively washed with DMSO:H_2O, water and KPB. With KPB serving as electrolyte, a CV was recorded with vertex potentials at −400 and 300 mV, and a scanning rate of 50 mV/sec to ensure the formation of a monolayer. Once that was established (data not shown), MBN in a final concentration of 100 nM was added to the cell, at an applied potential of 0 mV. The MBN nitrile stretch peak could be detected, and its growth was followed with time (see Figure S3, top). The peak remained after removing excess unbound MBN from the cell and washing it, indicating that it represents MBN covalently bound to the gold surface. Subsequently a potential series from −400 mV to +100 mV was applied, with SEIRA spectra being recorded at each potential (see main text). Then another identical CV was performed, to

ensure that the monolayer was still attached to the gold electrode, followed by a reductive potential of −1.5 V to remove the monolayer. Then another CV and SEIRA spectrum were recorded, to ascertain the desorption of the monolayer from the surface. (see Figure S3, bottom).

Figure S3. top: SEIRA spectra, showing the MBN peak after its addition to a SEIRA cell containing a Thiophenol monolayer (black, red, blue, teal and pink spectra taken at 2.5 min intervals after MBN addition to cell), after removing the MBN solution from the cell (green) and after applying −1.5 V to the WE. **Bottom:** Cyclic voltammograms performed on the Thiophenol:MBN mixed monolayer before (red) and after (blue) applying a potential series and after applying a voltage of −1.5 V (black).

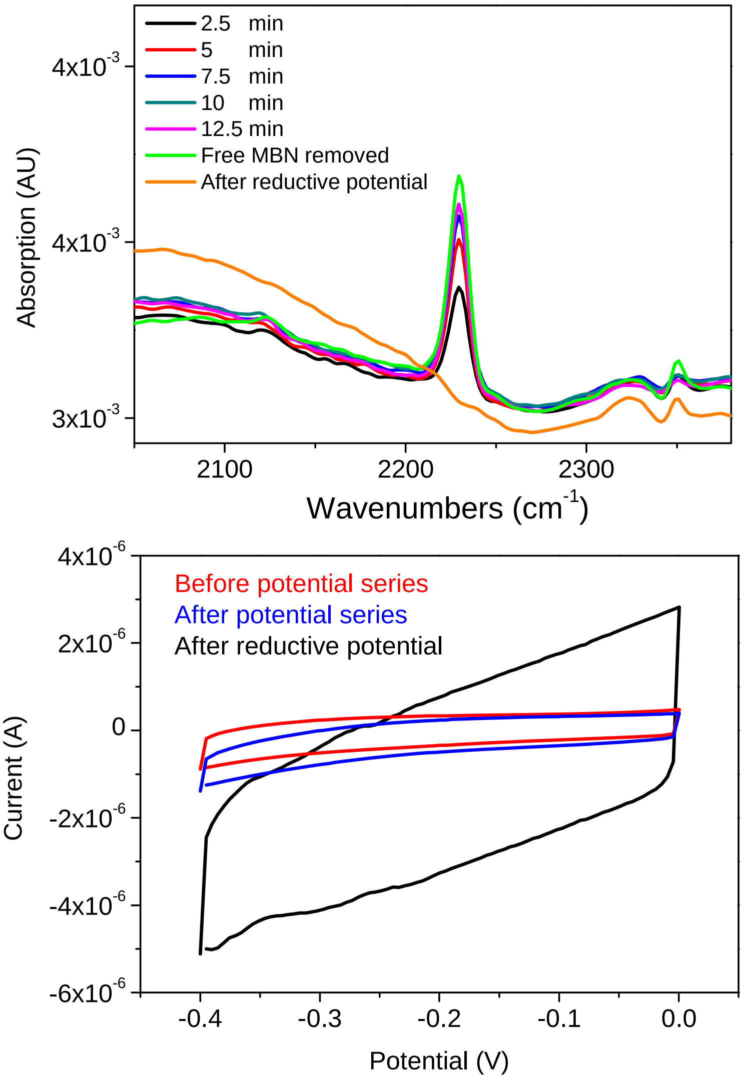

3. MBN-SAM on Ag Electrodes

Ag ring electrodes were mechanically polished, rinsed with ultra pure H_2O (R > 18 MΩ), and then immersed in 0.1 M KCl aqueous solution. After removing organic impurities by applying −2.0 V for 40 s, electrochemical surface roughening was achieved by multiple oxidation-reduction cycles at potentials of +0.3 V and −0.3 V respectively, followed by a potential of −0.3 V for 5 min. After washing with H_2O, the electrode was immersed in a 8 mM solution of MBN in DMSO:H_2O 3:1 (v/v)

at 4 °C for 18 h to form a homogeneous monolayer on the silver surface. After overnight incubation the electrode showed a CV typical of a SAM-coated metal electrode (see Figure S4), both before and after the series of applied potentials. After the potential series a reductive potential was applied to the electrode to remove the SAM, and a CV was recorded, featuring a much higher capacitance, due to the loss of the SAM (Figure S4).

Figure S4. Cyclic voltammograms of Ag working electrode in SERS setup, with MBN monolayer after applying a potential series (black) and after applying a reductive potential to remove the monolayer (red).

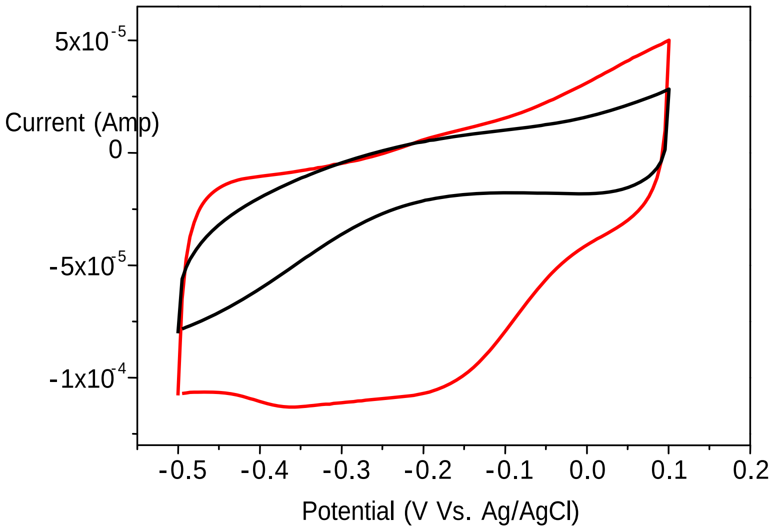

4. The Nitrile Stretching Frequency of MBN in an Aqueous Solution

To see what effect the buffer environment has on the nitrile stretching frequency of MBN, MBN dissolved in DMSO was subjected to a controlled increment of their aqueous phase content by titration with KPB, and changes to the nitrile stretch frequency were recorded (Figure S5).

MBN was dissolved in DMSO at 15 mM. IR transmission measurements of the resulting solution were performed in a split-beam cell for liquid samples (path length 25 µm) equipped with CaF_2 windows, using a Digilabs FTS-6000 spectrometer with a photoconductive MCT detector. The spectral resolution was 2 cm^{-1}. The spectrometer was purged with dry air. 1000–1500 scans were accumulated for each spectrum. The background used was pure DMSO. Three repetitions were made. Fractions of KPB, from 10% to 90% (v/v) were added to the two solutions, and IR spectra were recorded for each fraction, with KPB:DMSO in the corresponding ratio as background.

For MBN dissolved in 100% DMSO, the nitrile stretching frequency of 2227.7 ± 0.03 cm^{-1} has been observed, in agreement with Schkolnik *et al.* [2] and Suydam and Boxer [3]. Upon addition of KPB fractions of 0–30%, the frequency increased linearly. At 40% KPB the water insoluble MBN started precipitating, and from 50% on, the observed frequency was that of solid MBN, at 2225–2226 cm^{-1} (2226 cm^{-1} was recorded by Raman spectroscopy for solid MBN [2]). When fitting the linear portion of the titration curve (Figure S5b) and projecting the fit to 100% KPB, one arrives at 2233.6 cm^{-1} for the CN stretch in buffer-exposed MBN. This is a reasonable value at 6 cm^{-1} above

that measured in DMSO, which is in line with the blue-shift in the CN stretching mode due to H-bonding interactions documented previously [4–6]. This value is also similar to 2234 cm^{-1} measured for *p*-PheCN, a similar compound, in buffer by Fafarman and Boxer [6].

Figure S5. Titration of MBN 15 mM in DMSO with fractions (v/v) of KPB. **Left**: Titration curve. **Right**: A linear fit of the fractions 0-30%. Linear fit equation: ν = 2227.6 + 0.060x. R^2 = 0.911. Projection to 100% KPB: 2233.6 cm^{-1}.

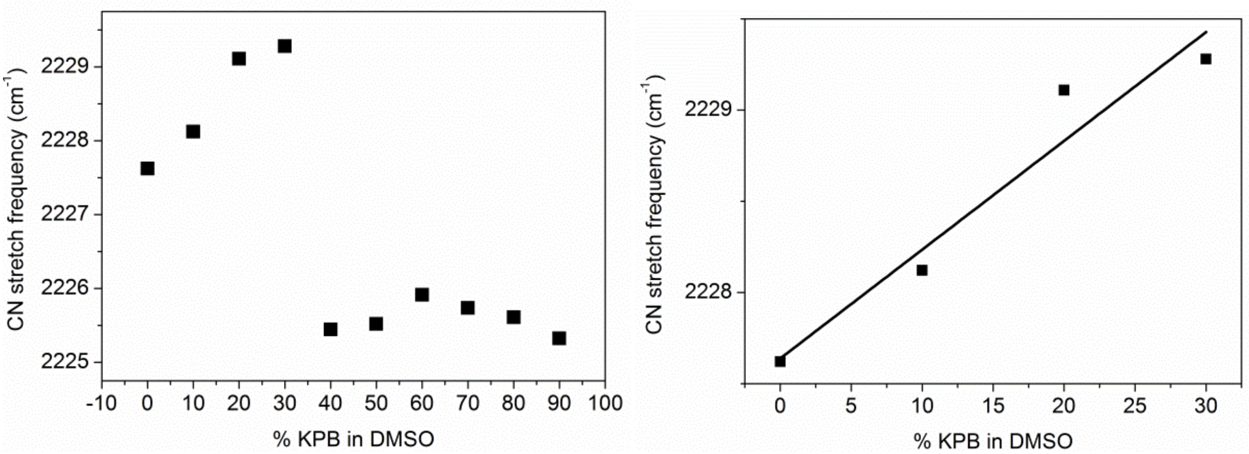

References

1. Smith, C.P.; Dwhite, H.S. Theory of the interfacial potential distribution and reversible voltammetric response of electrodes coated with electroactive molecular films. *Anal. Chem.* **1992**, *64*, 2398–2405.
2. Schkolnik, G.; Utesch, T.; Salewski, J.; Tenger, K.; Millo, D.; Kranich, A.; Zebger, I.; Schulz, C.; Zimányi, L.; Rákhely, G.; *et al*. Mapping local electric fields in proteins at biomimetic interfaces. *Chem. Commun.* **2012**, *48*, 70–72.
3. Suydam, I.T.; Boxer, S.G. Vibrational Stark effects calibrate the sensitivity of vibrational probes for electric fields in proteins. *Biochemistry* **2003**, *42*, 12050–12055.
4. Aschaffenburg, D.J.; Moog, R.S. Probing hydrogen bonding environments: Solvatochromic effects on the CN vibration of benzonitrile. *J. Phys. Chem. B* **2009**, *113*, 12736–12743.
5. Ghosh, A.; Remorino, A.; Tucker, M.J.; Hochstrasser, R.M. 2D IR photon echo spectroscopy reveals hydrogen bond dynamics of aromatic nitriles. *Chem. Phys. Lett.* **2009**, *469*, 325–330.
6. Fafarman, A.T.; Sigala, P.A.; Herschlag, D.; Boxer, S.G. Decomposition of vibrational shifts of nitriles into electrostatic and hydrogen-bonding effects. *J. Am. Chem. Soc.* **2010**, *132*, 12811–12813.
